# Crystal structures and Hirshfeld surface analyses of two new tetra­kis-substituted pyrazines and a degredation product

**DOI:** 10.1107/S2056989020002133

**Published:** 2020-02-18

**Authors:** Ana Tesouro Vallina, Helen Stoeckli-Evans

**Affiliations:** a Institute of Chemistry, University of Neuchâtel, Av. de Bellvaux 15, CH-2000 Neuchâtel, Switzerland; b Institute of Physics, University of Neuchâtel, rue Emile-Argand 11, CH-2000 Neuchâtel, Switzerland

**Keywords:** crystal structure, pyrazine, tetra­kis-substituted, C—H⋯π inter­actions, offset π–π inter­actions, Hirshfeld surface analysis

## Abstract

The two new tetra­kis-substituted pyrazines, 1,1′,1′′,1′′′-(pyrazine-2,3,5,6-tetra­yl) tetra­kis­(*N*,*N*-di­methyl­methanamine), C_16_H_32_N_6_, (I) and *N*,*N*′,*N*′′,*N*′′′-[pyrazine-2,3,5,6-tetra­yltetra­kis­(methyl­ene)]tetra­kis­(*N*-methyl­aniline), C_36_H_40_N_6_, (II), both crystallize with half a mol­ecule in the asymmetric unit; the whole mol­ecules are generated by inversion symmetry. There are weak intra­molecular C—H⋯N hydrogen bonds present in both mol­ecules and in (II) the pendant *N*-methyl­aniline rings are linked by a C—H⋯π inter­action. The degredation product, *N*,*N′-*[(6-phenyl-6,7-di­hydro-5*H*-pyrrolo­[3,4-*b*]pyrazine-2,3-di­yl)bis(methyl­ene)]bis­(*N*-methyl­aniline), C_28_H_29_N_5_, (III), was obtained several times by reacting (II) with different metal salts. Here, the 6-phenyl ring is almost coplanar with the planar pyrrolo­[3,4-*b*]pyrazine unit (r.m.s. deviation = 0.029 Å), with a dihedral angle of 4.41 (10)° between them. The two *N*-meth­yl­aniline rings are inclined to the planar pyrrolo­[3,4-*b*]pyrazine unit by 88.26 (10) and 89.71 (10)°, and to each other by 72.56 (13)°. There are also weak intra­molecular C—H⋯N hydrogen bonds present involving the pyrazine ring and the two *N*-methyl­aniline groups. In the crystal of (I), there are no significant inter­molecular contacts present, while in (II) mol­ecules are linked by a pair of C—H⋯π inter­actions, forming chains along the *c*-axis direction. In the crystal of (III), mol­ecules are linked by two pairs of C—H⋯π inter­actions, forming inversion dimers, which in turn are linked by offset π–π inter­actions [inter­centroid distance = 3.8492 (19) Å], forming ribbons along the *b*-axis direction.

## Abstract 2

The crystal structures of two new tetra­kis-substituted pyrazine compounds, 1,1′,1′′,1′′′-(pyrazine-2,3,5,6-tetra­yl)tetra­kis­(*N*,*N*-di­methyl­methanamine) and *N*,*N*′,*N*′′,*N*′′′-[pyrazine-2,3,5,6tetra­yltetra­kis­(methyl­ene)]tetra­kis­(*N*-methyl­aniline), and a degredation product, *N*,*N*′-[(6-phenyl-6,7-di­hydro-5*H*-pyrrolo­[3,4-*b*]pyrazine-2,3-di­yl)bis­(methyl­ene)]bis­(*N*-methyl­aniline), are described and have been analysed by Hirshfeld surface analysis.

## Chemical context   

Tetra­kis-substituted pyrazines, which are potential bis-tridentate ligands, have been used in coordination chemistry since the 1980′s, to form not only mononuclear and binuclear complexes but also multi-dimensional coordination polymers. A search of the Cambridge Structural Database (CSD, Version 5.41, last update November 2019; Groom *et al.*, 2016 ▸) reveals that the principal tetra­kis-substituted pyrazine ligands that have been used are 2,3,5,6-tetra­kis­(pyridin-2-yl)pyrazine, which was first synthesized by Goodwin & Lions (1959 ▸), and 2,3,5,6-pyrazine­tetra­carb­oxy­lic acid, which was first synthesized by Wolff at the end of the 19th century (Wolff, 1887 ▸, 1893 ▸). Since then the coordination chemistry of only a small number of tetra­kis-substituted pyrazines has been studied, for example tetra­kis­(amino­meth­yl)pyrazine (Ferigo *et al.*, 1994 ▸) and, more recently, the new ligand 2,3,5,6-tetra­kis­(4-carb­oxy­phen­yl) pyrazine, which has been shown to be extremely successful in forming metal–organic frameworks (Jiang *et al.*, 2017 ▸; Wang *et al.*, 2019 ▸).
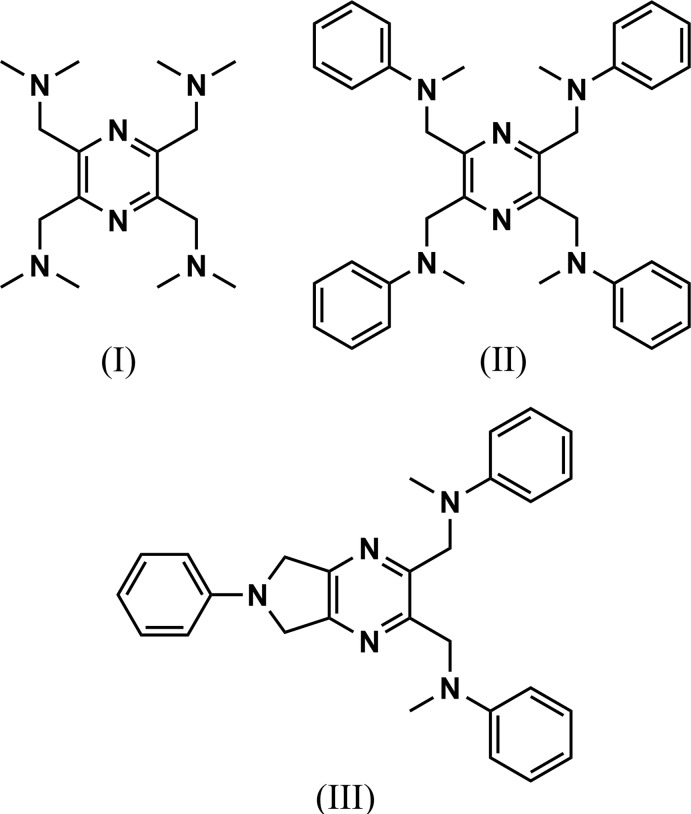


In our search for new tetra­kis-substituted pyrazine ligands (Tesouro Vallina, 2001 ▸), *viz*. potential bis-tridentate ligands, the title compounds, 1,1′,1′′,1′′′-(pyrazine-2,3,5,6-tetra­yl) tetra­kis­(*N*,*N*-di­methyl­methanamine) (I) and *N*,*N*′,*N*′′,*N*′′′-[pyrazine-2,3,5,6-tetra­yltetra­kis­(methyl­ene)]tetra­kis­(*N*-methyl­aniline) (II) were synthesized. During attempts to form transition-metal complexes of (II), the degradation product, *N*,*N*′-[(6-phenyl-6,7-di­hydro-5*H*-pyrrolo­[3,4-*b*]pyrazine-2,3-di­yl)bis­(methyl­ene)]bis­(*N*-methyl­aniline) (III) was often formed. Herein, we describe their mol­ecular and crystal structures, together with the Hirshfeld surface analysis of their crystal packing.

## Structural commentary   

**Figure 1:**
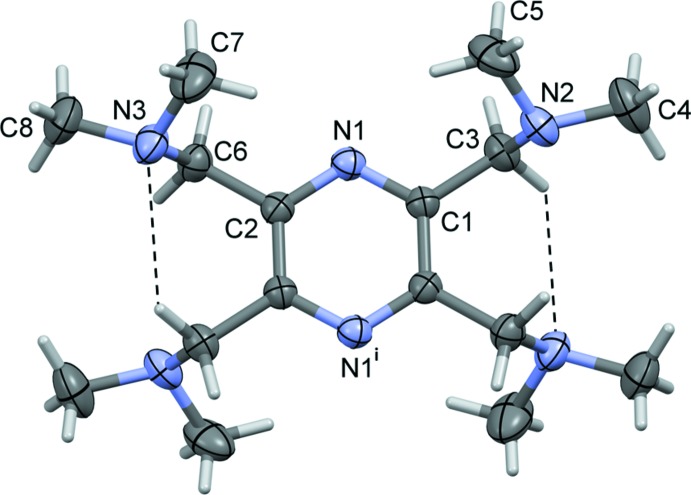
A view of the mol­ecular structure of compound (I), with atom labelling [symmetry code: (i) −*x*, −*y*, −*z* + 1]. Displacement ellipsoids are drawn at the 30% probability level. Intra­molecular C—H⋯N inter­actions (Table 1 ▸) are shown as dashed lines.

The mol­ecular structure of compound (I) is illustrated in Fig. 1 ▸. The mol­ecule possesses inversion symmetry with the pyrazine ring being located about a center of symmetry. The adjacent di­methyl­methanamine substituents, in positions 2,3 (and 5,6), are directed above and below the plane of the pyrazine ring. There is a short intra­molecular C3—H3*A*⋯N3^i^ contact on either side of the mol­ecule [symmetry code: (i) −*x*, −*y*, −*z* 1 ], linking the two di­methyl­methanamine substituents (Fig. 1 ▸ and Table 1 ▸).

**Figure 2:**
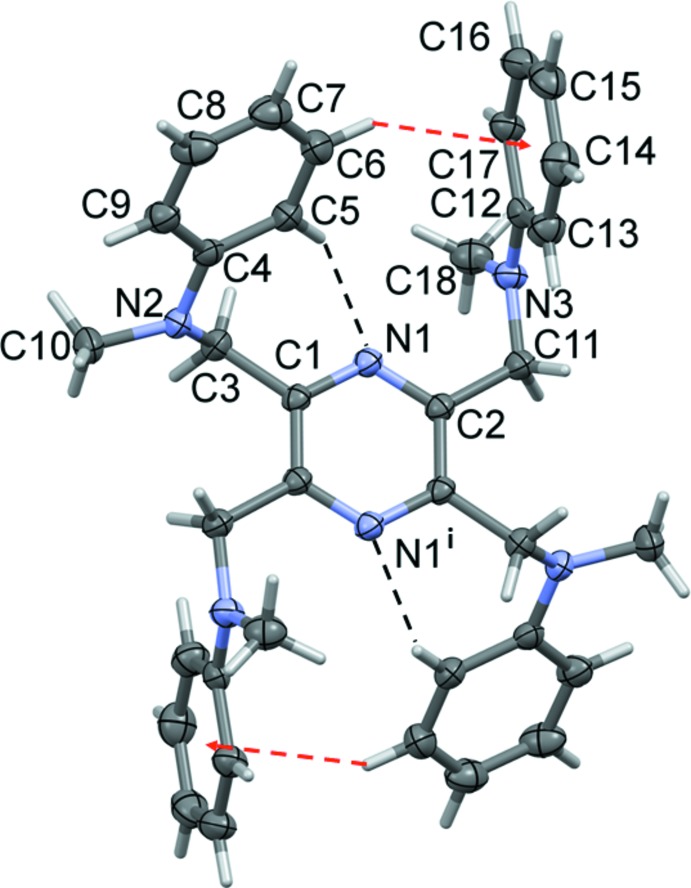
A view of the mol­ecular structure of compound (II), with atom labelling [symmetry code: (i) −*x* + 1, −*y* + 1, −*z* + 2]. Displacement ellipsoids are drawn at the 30% probability level. Intra­molecular C—H⋯N inter­actions (Table 2 ▸) are shown as dashed lines and the intra­molecular C—H⋯π inter­actions (Table 2 ▸) as red dashed arrows.

The mol­ecular structure of compound (II) is illustrated in Fig. 2 ▸. This mol­ecule also possesses inversion symmetry with the pyrazine ring being located about a center of symmetry. Again the adjacent methyl­aniline substituents, in positions 2,3 (and 5,6), are directed above and below the plane of the pyrazine ring. Rings C4–C9 and C12–C17 are inclined to the pyrazine ring by 63.62 (10) and 86.83 (10)°, respectively, and to each other by 78.28 (11)°. There are short intra­molecular C5—H5⋯N1 contacts on either side of the mol­ecule involving a methyl­aniline ring and the adjacent pyrazine N atom, and the methyl­aniline substituents in positions 2,6 (and 3,5) are linked by an intra­molecular C6—H6⋯π inter­action (Fig. 2 ▸ and Table 2 ▸).

**Figure 3:**
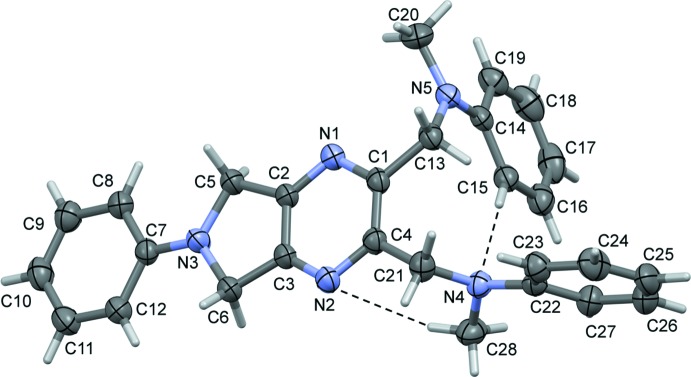
A view of the mol­ecular structure of compound (III), with atom labelling. Displacement ellipsoids are drawn at the 30% probability level. Intra­molecular C—H⋯N inter­actions (Table 3 ▸) are shown as dashed lines.

The mol­ecular structure of compound (III) is illustrated in Fig. 3 ▸. One side of the mol­ecule has been transformed into a pyrrolo unit fused to the pyrazine ring. The 6-phenyl ring (C7–C12) is almost coplanar with the planar pyrrolo­[3,4-*b*]pyrazine unit (N1–N3/C1–C6; r.m.s. deviation = 0.029 Å), forming a dihedral angle of 4.41 (10)°. On the other side of the mol­ecule, the two adjacent *N*-methyl­aniline rings (C14–C19 and C22–C27) are inclined to the planar pyrrolo­[3,4-*b*]pyrazine unit by 88.26 (10) and 89.71 (10)°, and to each other by 72.56 (13)°. There are also weak intra­molecular C—H⋯N hydrogen bonds present involving the pyrazine ring and the two *N*-methyl­aniline groups (Fig. 3 ▸ and Table 3 ▸).

## Supra­molecular features   

**Figure 4:**
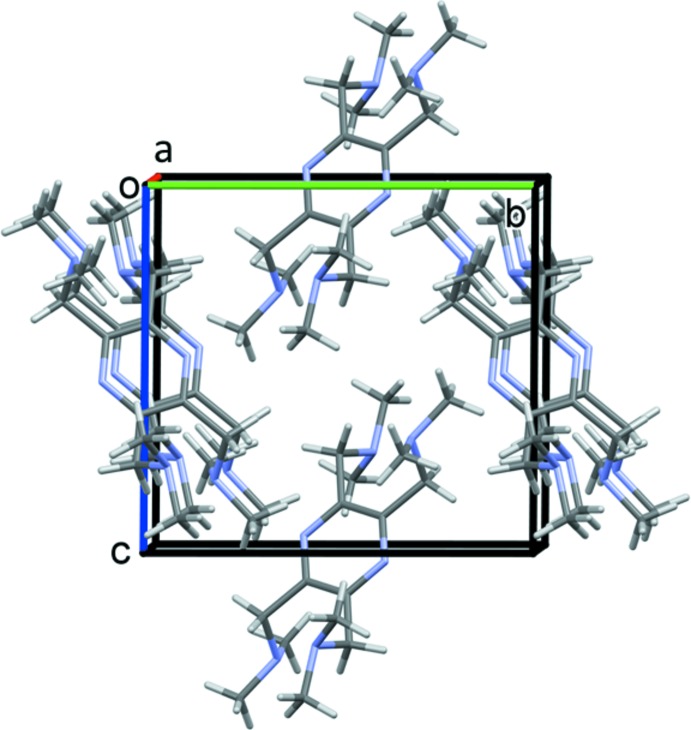
A view along the *a* axis of the crystal packing of compound (I).

In the crystal of (I), there are no significant inter­molecular inter­actions present (Fig. 4 ▸).

**Figure 5:**
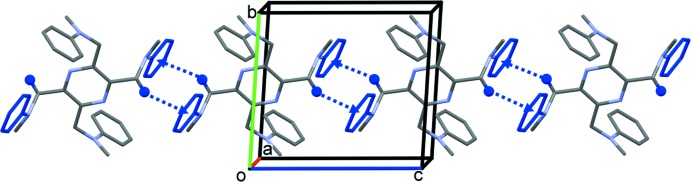
A view along the *a* axis of the crystal packing of compound (II). The C3—H3*A*⋯π inter­actions (Table 2 ▸) are shown as blue dashed arrows, and for clarity, only H atom H3*A* (blue) has been included.

In the crystal of (II), mol­ecules are linked by a pair of C—H⋯π inter­actions, forming chains that propagate along the [001] direction (Fig. 5 ▸ and Table 2 ▸).

**Figure 6:**
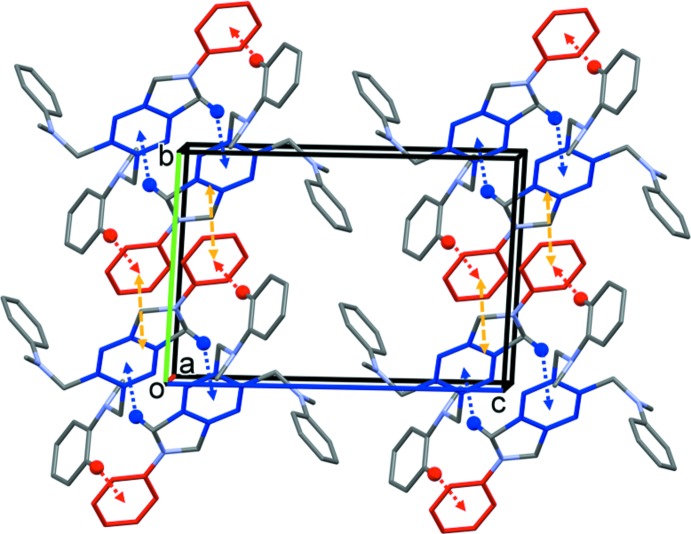
A view along the *a* axis of the crystal packing of compound (III). The C—H⋯π inter­actions (Table 3 ▸) are shown as blue and red dashed arrows. For clarity, only the H atoms H6*B* (blue) and H23 (red) have been included. The offset π-π- inter­actions are shown as orange dashed double arrows.

In the crystal of (III), mol­ecules are linked by two pairs of C—H⋯π inter­actions, forming inversion dimers. Offset π–π inter­actions link the dimers to form ribbons propagating along the [010] direction; see Fig. 6 ▸ and Table 3 ▸. The offset π–π inter­action, C*g*3⋯C*g*6^ii^, where C*g*3 and C*g*6 are, respectively, the centroids of the phenyl ring (C7–C12) and the pyrrolo[3,4-*b*]pyrazine ring system, has a centroid–centroid distance of 3.8492 (14) Å, α = 4.41 (10)°, inter­planar distances of 3.6495 (14) and 3.5490 (7) Å, with an offset of 1.49 Å [symmetry code: (ii) −*x* + 1, −*y* + 1, −*z*].

## Hirshfeld surface analysis and two-dimensional fingerprint plots   

The Hirshfeld surface analysis (Spackman & Jayatilaka, 2009 ▸) and the associated two-dimensional fingerprint plots (McKinnon *et al.*, 2007 ▸) were performed with *CrystalExplorer17* (Turner *et al.*, 2017 ▸).

**Figure 7:**
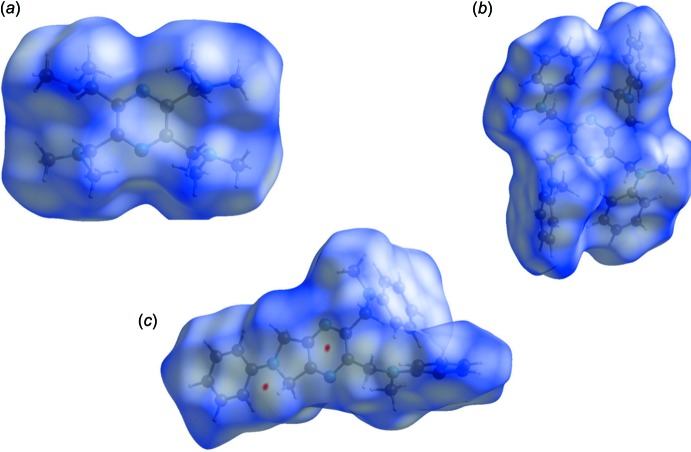
(*a*) The Hirshfeld surface of compound (I), mapped over *d*_norm_ in the colour range −0.7519 to 1.6997 a.u., (*b*) the Hirshfeld surface of compound (II), mapped over *d*_norm_ in the colour range −0.7519 to 1.6997 a.u. and (*c*) the Hirshfeld surface of compound (III), mapped over *d*_norm_ in the colour range −0.7519 to 1.6997 a.u..

The Hirshfeld surfaces are colour-mapped with the normalized contact distance, *d*_norm_, from red (distances shorter than the sum of the van der Waals radii) through white to blue (distances longer than the sum of the van der Waals radii). The Hirshfeld surfaces (HS) of the title compounds, mapped over *d*_norm_, are given in Fig. 7 ▸. It is evident from Figs. 7 ▸ *a* and 7*b* that there are no contact distances shorter than the sum of the van der Waals radii in the crystals of either compounds (I) or (II). For compound (III) (Fig. 7 ▸ *c*), two small red spots indicate the presence of weak C⋯H contacts (see Table 3 ▸).

**Figure 8:**
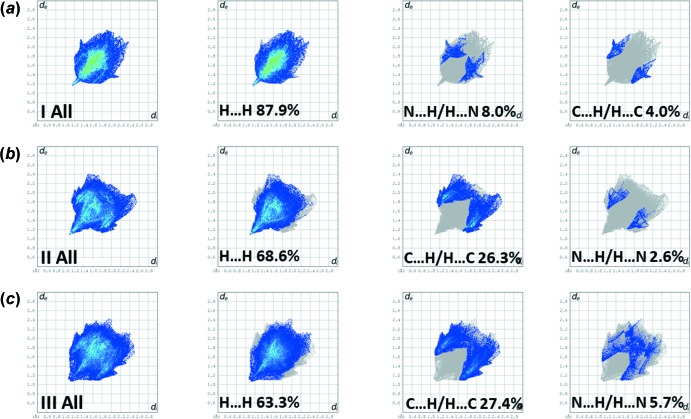
(*a*) The full two-dimensional fingerprint plot for compound (I), and fingerprint plots delineated into H⋯H, N⋯H/H⋯N and C⋯H/H⋯C contacts, (*b*) the full two-dimensional fingerprint plot for compound (II), and fingerprint plots delineated into H⋯H, C⋯H/H⋯C and N⋯H/H⋯N contacts and (*c*) the full two-dimensional fingerprint plot for compound (III), and fingerprint plots delineated into H⋯H, C⋯H/H⋯C and N⋯H/H⋯N contacts.

The two-dimensional fingerprint plots for the title compounds are given in Fig. 8 ▸. They reveal, as expected, that the principal contributions to the overall surface involve H⋯H contacts at 87.9% for (I) (Fig. 8 ▸ *a*), 68.6% for (II) (Fig. 8 ▸ *b*), and 63.3% for (III) (Fig. 8 ▸ *c*). The second most important contribution to the HS for compound (I) is from the N⋯H/H⋯N contacts at 8.0%; for compounds (II) and (III) the second most significant contributions are from the C⋯H/H⋯C contacts at 26.3 and 27.4%, respectively. For compound (I), the third most important contribution to the HS is from the C⋯H/H⋯C contacts at 4.0%, while for compounds (II) and (III) it is from the N⋯H/H⋯N contacts at 2.6 and 5.7%, respectively. All other atom⋯atom contacts contribute less that 2% to the HS for all three compounds.

## Database survey   

A search of the CSD (Version 5.41, last update November 2019; Groom *et al.*, 2016 ▸) for the structure of 2,3,5,6-tetra­kis­(pyridin-2-yl)pyrazine gave 289 hits, of which 91 structures are polymeric. The first polymeric compound to be reported in 1995 was for a trinuclear cobalt(II) one-dimensional coordination polymer, *catena*-[bis­(μ_2_-chloro)­aceto­nitrile­tetra­chloro-[2,3,5,6-tetra­kis­(2-pyrid­yl)pyrazine]­tricobalt(II)] (CSD ref­code TUPWAC; Constable *et al.*, 1995 ▸).

A search for the structure of 2,3,5,6-pyrazine­tetra­carb­oxy­lic acid gave 92 hits, of which 64 are polymeric. Here, the first polymeric compound to be reported in 1986 was for a binuclear iron(II) polymer chain, *catena*-[μ_2_-(2,5-di­carb­oxy­pyrazine-3,6-di­carboxyl­ato-*N*,*O*)*trans*-di­aqua­diiron(II)] dihydrate (DUWROC; Marioni *et al.*, 1986 ▸).

A search for the structure of tetra­kis­(amino­meth­yl)pyrazine yielded only eight hits, of which five compounds are polymeric; see for example *catena*-[μ_2_-[tetra­kis­(amino­meth­yl)pyrazine-*N*,*N*′,*N*′′]manganese dichloride dihydrate] (PITXEV; Ferigo *et al.*, 1994 ▸), and *catena*-[[μ_2_-2,3,5,6-tetra­kis­(amino­meth­yl)pyrazine]­bis­(μ_2_-chloro)­dichloro­dicopper hydrate] (PITXIZ; Ferigo *et al.*, 1994 ▸).

Recently a new ligand, 2,3,5,6-tetra­kis­(4-carb­oxy­phenyl pyrazine), has been shown to be extremely successful in forming 17 metal–organic frameworks (MOFs). It was designed by Jiang and coworkers (Jiang *et al.*, 2017 ▸) who produced the first MOF using this ligand, *viz. catena*-[(μ-4,4′,4′′,4′′′-pyrazine-2,3,5,6-tetra­benzoato)bis­(*N*,*N*-di­methyl­formamide)­dizinc unknown solvate] (NAWXER; Jiang *et al.*, 2017 ▸). Since then the ligand has been used by a number of groups, and the most recent MOF to be published is *catena*-[(μ-4,4′-bi­pyridine)­bis­(μ-hydroxo)bis­[μ-di­hydrogen 4,4′,4′′,4′′′-(pyrazine-2,3,5,6-tetra­yl)tetra­benzoato]trinickel unknown solvate] (HOQTUF; Wang *et al.*, 2019 ▸).

In relation to the structure of compound (III), a search for the substructure pyrrolo­[3,4-*b*]pyrazine yielded only two hits. They concern di­pyrrolo­[3,4-*b*:3′,4′-*e*]pyrazine structures that possess inversion symmetry, *viz*. 2,6-dibenzyl-1,2,3,5,6,7-hexa­hydro­dipyrrolo­[3,4-*b*:3′,4′-*e*]pyrazine (EXUHIO; Gasser & Stoeckli-Evans, 2004 ▸) and 2,6-bis­(4-meth­oxy­benz­yl)-1,2,3,5,6,7-hexa­hydro­dipyrrolo­[3,4-*b*:3′,4′-*e*]pyrazine (EXU­HOU; Gasser & Stoeckli-Evans, 2004 ▸). They were prepared during attempts to form 1,2,3,5,6,7-hexa­hydro-2,4,6,8-tetra­aza-*s*-indacene by reacting 2,3,5,6-tetra­kis­(bromo­meth­yl)pyrazine (Ferigo *et al.*, 1994 ▸; TOJXUN: Assoumatine & Stoeckli-Evans, 2014 ▸) with the corresponding amines. In contrast to (III), where the pyrrolo ring is planar (r.m.s. deviation = 0.029 Å) and inclined by only 2.00 (12)° to the pyrazine ring, here the pyrrolo groups have envelope conformations with the pyrrolo N atoms as the flaps. Their mean planes are inclined to the pyrazine ring by 7.88 (16)° in EXUHIO and by 8.05 (7)° in EXUHOU.

## Synthesis and crystallization   

**Synthesis of 1,1′,1′′,1′′′-(pyrazine-2,3,5,6-tetra­yl) tetra­kis­(** ***N***,***N*** **-di­methyl­methanamine) (I):**

A large excess of dimethyl amine hydro­chloride in water was neutralized with NaOH in an ice bath. Me_2_NH formed *in situ* as a gas and was directly condensed in a round-bottom flask in an acetone/liquid N_2_ bath at about 213 K using a weak vacuum. Once a sufficient qu­antity of liquid amine had formed, a solution of 2,3,5,6-tetra­kis­(bromo­meth­yl)pyrazine (0.4530 g, 1 mmol) in 50 ml of CH_2_Cl_2_ was added dropwise at low temperature (*ca* 243 K). The reaction was left for about 4 h, allowing the temperature rise to RT. The excess amine was allowed to evaporate off before the solvent was gassed off. The residue obtained was dissolved in 40 ml of MeOH and passed through a resin column (15 g of Dowex 1 X8) previously charged with OH^−^ ions in order to exchange the HBr mol­ecules, still attached to the ligand, by H_2_O mol­ecules. About 150 ml were used as eluent. Solvent evaporation yielded 0.27 g (87%) of a light-yellow powder of compound (I). Colourless block-like crystals were obtained by slow diffusion of hexane into a solution of the ligand in di­chloro­methane.

^1^H NMR (CDCl_3_, 200 MHz, ppm): 3.65 (*s*, 8H, CH_2_), 2.15 (*s*, 12H, CH_3_). ^13^C NMR (D_2_O, 400 MHz, ppm): 152.16, 62.53, 46.54. IR (KBr pellet, cm^−1^): 2974 (*s*), 2942 (*s*), 2854 (*m*), 2820 (*vs*), 2772 (*vs*), 1635 (*b*), 1456 (*s*), 1414 (*m*), 1348 (*s*), 1259 (*s*), 1204 (*m*), 1168 (*m*), 1027 (*vs*), 987 (*m*), 841 (*s*). MS (EI, 70 eV), m/z: 310 (MH^+^), 264, 178. Anal. for C_16_H_32_N_6_ (308.5 g mol^−1^) Calculated (%) C 62.30, H 10.46, N 27.24. Found (%) C 61.86, H 10.73, N 27.50.

**Synthesis of** ***N***,***N*** **′**,***N*** **′′**,***N*** **′′′-[pyrazine-2,3,5,6-tetra­yltetra­kis(methyl­ene)]tetra­kis­(** ***N*** **-methyl­aniline) (II)**:

A solution of 2,3,5,6-tetra­kis­(bromo­meth­yl)pyrazine (0.4530 g, 1 mmol) in 35 ml of CH_3_CN was added dropwise to a suspension of *N*-methyl­aniline (1.2 ml, 10 mmol) and Na_2_CO_3_ (5.3 g, 50 mmol) in 25 ml of CH_3_CN. The colour changed immediately from light to deep yellow. The mixture was refluxed for *ca* 2 h, followed by TLC and then cooled to RT. The white precipitate (NaBr and excess Na_2_CO_3_) was filtered off and the filtrate was evaporated under vacuum. The residue was dissolved in hexane and the insoluble yellow powder obtained was recovered, washed with more hexane and then dried to yield 0.335 g (60%) of compound (II). Pale-greenish-yellow block-like crystals were obtained by slow evaporation of a CDCl_3_ solution of (II) in an NMR tube.

^1^H NMR (CDCl_3_, 200 MHz, ppm): 7.14 (*t*, 8H, ph), 6.68 (*m*, 12H, ph), 4.58 (*s*, 8H, CH_2_), 2.79 (*s*, 12H, CH_3_). ^13^C NMR (CD_3_OD, 400 MHz, ppm): 149.64, 149.31, 128.94, 116.92, 113.17, 54.75, 39.46. IR (KBr pellet, cm^−1^): 2926 (*w*), 1601 (*s*), 1508 (*vs*), 1446 (*m*), 1377 (*m*), 1366 (*m*), 1313 (*m*), 1257 (*m*), 1212 (*m*), 1117 (*w*), 993 (*w*), 820 (*w*), 745 (*s*), 689 (*m*). MS (EI, 70 eV), *m*/*z*: 594 (*M*K^+^), 374, 291. Analysis for C_36_H_40_N_6_ (556.7 g mol^−1^) Calculated (%) C 77.66, H 7.24, N 15.09. Found (%) C 76.82, H 7.19, N 15.07.

**Synthesis of** ***N***,***N*** **′-[(6-phenyl-6,7-di­hydro-5** ***H*** **-pyrrolo[3,4-** ***b*** **]pyrazine-2,3-di­yl)bis­(methyl­ene)]bis­(** ***N*** **-methyl­aniline) (III):**

Hexagonal pale-yellow plate-like crystals of compound (III) were obtained several times when reacting (II) with different metal salts, such as Zn(ClO_4_)_2_ (in excess), MnCl_2_·4H_2_O and Ni(AcO)_2_·4H_2_O. No spectroscopic or other analytical data are available for this compound.

## Refinement   

Crystal data, data collection and structure refinement details are summarized in Table 4 ▸. The C-bound H atoms were included in calculated positions and treated as riding on their parent C atom: C—H = 0.93–0.97 Å with *U*_iso_(H) =1.5*U*_eq_(C-meth­yl) and 1.2*U*_eq_(C) for other H atoms. Note for compound (III): using the Stoe IPDS I, a one-circle diffractometer, to measure data for the triclinic system often only 93% of the Ewald sphere is accessible. Hence, the diffrn_reflns_Laue_measured_fraction_full of 0.939 is below the required minimum of 0.95.

## Supplementary Material

Crystal structure: contains datablock(s) I, II, III, Global. DOI: 10.1107/S2056989020002133/xi2023sup1.cif

Structure factors: contains datablock(s) I. DOI: 10.1107/S2056989020002133/xi2023Isup2.hkl

Structure factors: contains datablock(s) II. DOI: 10.1107/S2056989020002133/xi2023IIsup3.hkl

Structure factors: contains datablock(s) III. DOI: 10.1107/S2056989020002133/xi2023IIIsup4.hkl

Supporting information file. DOI: 10.1107/S2056989020002133/xi2023IIsup5.cml

Supporting information file. DOI: 10.1107/S2056989020002133/xi2023IIIsup6.cml

CCDC references: 1984024, 1984023, 1984022

Additional supporting information:  crystallographic information; 3D view; checkCIF report

## Figures and Tables

**Table 1:** Hydrogen-bond geometry (Å, °) for (I)

*D*—H⋯*A*	*D*—H	H⋯*A*	*D*⋯*A*	*D*—H⋯*A*
C3—H3*A*⋯N3^i^	0.97	2.62	3.261 (4)	124

Symmetry code: (i) 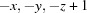.

**Table 2:** Hydrogen-bond geometry (Å, °) for (II) *Cg*2 and *Cg*3 are the centroids of rings C4–C9 and C12–C17, respectively.

*D*—H⋯*A*	*D*—H	H⋯*A*	*D*⋯*A*	*D*—H⋯*A*
C5—H5⋯N1	0.93	2.50	3.331 (3)	149
C6—H6⋯*Cg*3	0.93	2.99	3.804 (3)	147
C3—H3*A*⋯C*g*2^i^	0.97	2.83	3.561 (2)	133

Symmetry code: (i) 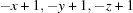.

**Table 3:** Hydrogen-bond geometry (Å, °) for (III) *Cg*2 and *Cg*3 are the centroids of rings N1/N2/C1–C4 and C7–C12, respectively.

*D*—H⋯*A*	*D*—H	H⋯*A*	*D*⋯*A*	*D*—H⋯*A*
C15—H15⋯N4	0.93	2.61	3.542 (3)	175
C28—H28*B*⋯N2	0.96	2.59	3.323 (3)	133
C6—H6*B*⋯*Cg*2^i^	0.97	2.82	3.601 (2)	138
C23—H23⋯*Cg*3^i^	0.93	2.97	3.881 (3)	168

Symmetry code: (i) 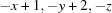.

**Table 4:** Experimental details

	(I)	(II)	(III)
Crystal data			
Chemical formula	C_16_H_32_N_6_	C_36_H_40_N_6_	C_28_H_29_N_5_
*M*_r_	308.47	556.74	435.56
Crystal system, space group	Monoclinic, *P*2_1_/*n*	Triclinic, *P* 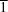	Triclinic, *P* 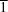
Temperature (K)	293	293	293
*a*, *b*, *c* (Å)	9.7577 (14), 10.348 (2), 9.9118 (16)	8.6753 (10), 8.9160 (11), 10.0631 (10)	8.686 (1), 9.7731 (11), 14.3948 (16)
α, β, γ (°)	90, 101.663 (15), 90	85.774 (10), 73.468 (11), 82.467 (11)	85.915 (13), 75.349 (13), 78.891 (13)
*V* (Å^3^)	980.2 (3)	739.21 (15)	1159.8 (2)
*Z*	2	1	2
Radiation type	Mo *K*α	Mo *K*α	Mo *K*α
μ (mm^−1^)	0.07	0.08	0.08
Crystal size (mm)	0.53 × 0.53 × 0.26	0.38 × 0.30 × 0.27	0.45 × 0.35 × 0.10

Data collection			
Diffractometer	Stoe–Siemens AED2, 4-circle	Stoe–Siemens AED2, 4-circle	Stoe *IPDS* 1
No. of measured, independent and observed [*I* > 2σ(*I*)] reflections	3347, 1818, 1111	5354, 2741, 1913	8653, 3953, 1518
*R*_int_	0.055	0.031	0.051
(sin θ/λ)_max_ (Å^−1^)	0.605	0.605	0.600

Refinement			
*R*[*F* ^2^ > 2σ(*F* ^2^)], *wR*(*F* ^2^), *S*	0.060, 0.154, 1.10	0.049, 0.108, 1.12	0.035, 0.079, 0.68
No. of reflections	1818	2741	3953
No. of parameters	105	193	301
H-atom treatment	H-atom parameters constrained	H-atom parameters constrained	H-atom parameters constrained
Δρ_max_, Δρ_min_ (e Å^−3^)	0.15, −0.12	0.13, −0.15	0.12, −0.12

Computer programs: *STADI4* (Stoe & Cie, 1997 ▸), *EXPOSE*, *CELL* and *INTEGRATE* in *IPDS-I* (Stoe & Cie, 2004 ▸), *X-RED* (Stoe & Cie, 1997 ▸), *SHELXS97* (Sheldrick, 2008 ▸), *Mercury* (Macrae *et al.*, 2020 ▸), *SHELXL2018/3* (Sheldrick, 2015 ▸), *PLATON* (Spek, 2020 ▸) and *publCIF* (Westrip, 2010 ▸).

## supplementary crystallographic information

### 1,1',1'',1'''-(Pyrazine-2,3,5,6-tetrayl)tetrakis(N,N-dimethylmethanamine) (I) Crystal data

**Table:**

C_16_H_32_N_6_	*F*(000) = 340
*M**_r_* = 308.47	*D*_x_ = 1.045 Mg m^−^^3^
Monoclinic, *P*2_1_/*n*	Mo *K*α radiation, λ = 0.71073 Å
*a* = 9.7577 (14) Å	Cell parameters from 22 reflections
*b* = 10.348 (2) Å	θ = 12.6–18.1°
*c* = 9.9118 (16) Å	µ = 0.07 mm^−^^1^
β = 101.663 (15)°	*T* = 293 K
*V* = 980.2 (3) Å^3^	Block, colourless
*Z* = 2	0.53 × 0.53 × 0.26 mm

### 1,1',1'',1'''-(Pyrazine-2,3,5,6-tetrayl)tetrakis(N,N-dimethylmethanamine) (I) Data collection

**Table:**

Stoe–Siemens AED2, 4-circle diffractometer	*R*_int_ = 0.055
Radiation source: fine-focus sealed tube	θ_max_ = 25.5°, θ_min_ = 2.7°
Plane graphite monochromator	*h* = −11→11
ω/\2q scans	*k* = 0→12
3347 measured reflections	*l* = −11→11
1818 independent reflections	2 standard reflections every 60 min
1111 reflections with *I* > 2σ(*I*)	intensity decay: 1%

### 1,1',1'',1'''-(Pyrazine-2,3,5,6-tetrayl)tetrakis(N,N-dimethylmethanamine) (I) Refinement

**Table:**

Refinement on *F*^2^	Secondary atom site location: difference Fourier map
Least-squares matrix: full	Hydrogen site location: inferred from neighbouring sites
*R*[*F*^2^ > 2σ(*F*^2^)] = 0.060	H-atom parameters constrained
*wR*(*F*^2^) = 0.154	*w* = 1/[σ^2^(*F*_o_^2^) + (0.0488*P*)^2^ + 0.2847*P*] where *P* = (*F*_o_^2^ + 2*F*_c_^2^)/3
*S* = 1.10	(Δ/σ)_max_ < 0.001
1818 reflections	Δρ_max_ = 0.15 e Å^−^^3^
105 parameters	Δρ_min_ = −0.12 e Å^−^^3^
0 restraints	Extinction correction: (*SHELXL2018/3*; Sheldrick, 2015), Fc^*^=kFc[1+0.001xFc^2^λ^3^/sin(2θ)]^-1/4^
Primary atom site location: structure-invariant direct methods	Extinction coefficient: 0.043 (7)

### 1,1',1'',1'''-(Pyrazine-2,3,5,6-tetrayl)tetrakis(N,N-dimethylmethanamine) (I) Special details

**Table:**

Geometry. All esds (except the esd in the dihedral angle between two l.s. planes) are estimated using the full covariance matrix. The cell esds are taken into account individually in the estimation of esds in distances, angles and torsion angles; correlations between esds in cell parameters are only used when they are defined by crystal symmetry. An approximate (isotropic) treatment of cell esds is used for estimating esds involving l.s. planes.

### 1,1',1'',1'''-(Pyrazine-2,3,5,6-tetrayl)tetrakis(N,N-dimethylmethanamine) (I) Fractional atomic coordinates and isotropic or equivalent isotropic displacement parameters (Å^2^)

**Table:**

	*x*	*y*	*z*	*U*_iso_*/*U*_eq_	
N1	0.07379 (18)	0.10665 (19)	0.46684 (19)	0.0504 (5)	
N2	0.2065 (2)	−0.0663 (2)	0.2414 (2)	0.0657 (7)	
N3	0.1836 (2)	0.1754 (2)	0.8033 (2)	0.0699 (7)	
C1	0.0393 (2)	0.0085 (2)	0.3779 (2)	0.0465 (6)	
C2	0.0345 (2)	0.0981 (2)	0.5885 (2)	0.0476 (6)	
C3	0.0864 (3)	0.0171 (3)	0.2426 (2)	0.0576 (7)	
H3A	0.009971	−0.008061	0.168567	0.069*	
H3B	0.111131	0.105746	0.226663	0.069*	
C4	0.2207 (4)	−0.0957 (4)	0.1004 (3)	0.1046 (12)	
H4A	0.234545	−0.016958	0.053752	0.157*	
H4B	0.137243	−0.137698	0.052395	0.157*	
H4C	0.299513	−0.151713	0.102710	0.157*	
C5	0.3335 (3)	−0.0092 (4)	0.3188 (3)	0.0979 (12)	
H5C	0.409862	−0.068297	0.321975	0.147*	
H5B	0.322027	0.009064	0.410892	0.147*	
H5A	0.352858	0.069624	0.275133	0.147*	
C6	0.0739 (3)	0.2095 (2)	0.6863 (3)	0.0615 (7)	
H6A	0.105348	0.281127	0.637289	0.074*	
H6B	−0.008203	0.237656	0.719505	0.074*	
C7	0.3174 (3)	0.1604 (4)	0.7623 (4)	0.1168 (15)	
H7A	0.346508	0.242054	0.731787	0.175*	
H7B	0.308288	0.098709	0.688686	0.175*	
H7C	0.386012	0.130348	0.839465	0.175*	
C8	0.1949 (4)	0.2720 (3)	0.9118 (3)	0.1000 (12)	
H8A	0.107852	0.277166	0.942547	0.150*	
H8B	0.215925	0.354560	0.876707	0.150*	
H8C	0.268255	0.248005	0.987590	0.150*	

### 1,1',1'',1'''-(Pyrazine-2,3,5,6-tetrayl)tetrakis(N,N-dimethylmethanamine) (I) Atomic displacement parameters (Å^2^)

**Table:**

	*U*^11^	*U*^22^	*U*^33^	*U*^12^	*U*^13^	*U*^23^
N1	0.0471 (11)	0.0513 (12)	0.0535 (12)	0.0003 (9)	0.0121 (9)	0.0005 (10)
N2	0.0654 (14)	0.0752 (16)	0.0629 (14)	0.0117 (12)	0.0279 (11)	0.0066 (12)
N3	0.0630 (14)	0.0758 (16)	0.0663 (14)	−0.0068 (12)	0.0021 (11)	−0.0189 (12)
C1	0.0433 (12)	0.0490 (14)	0.0465 (13)	0.0037 (11)	0.0076 (10)	0.0006 (12)
C2	0.0420 (12)	0.0478 (14)	0.0522 (14)	0.0030 (11)	0.0076 (10)	−0.0032 (11)
C3	0.0597 (15)	0.0629 (17)	0.0520 (14)	0.0059 (13)	0.0152 (12)	0.0041 (12)
C4	0.130 (3)	0.117 (3)	0.081 (2)	0.032 (2)	0.056 (2)	0.002 (2)
C5	0.0565 (17)	0.145 (3)	0.096 (2)	0.001 (2)	0.0259 (17)	0.019 (2)
C6	0.0660 (17)	0.0529 (15)	0.0666 (16)	−0.0012 (13)	0.0157 (13)	−0.0089 (13)
C7	0.060 (2)	0.154 (4)	0.131 (3)	−0.008 (2)	0.0089 (19)	−0.058 (3)
C8	0.105 (3)	0.111 (3)	0.078 (2)	−0.022 (2)	0.0064 (19)	−0.037 (2)

### 1,1',1'',1'''-(Pyrazine-2,3,5,6-tetrayl)tetrakis(N,N-dimethylmethanamine) (I) Geometric parameters (Å, º)

**Table:**

N1—C2	1.340 (3)	C4—H4B	0.9600
N1—C1	1.342 (3)	C4—H4C	0.9600
N2—C5	1.446 (4)	C5—H5C	0.9600
N2—C3	1.457 (3)	C5—H5B	0.9600
N2—C4	1.463 (3)	C5—H5A	0.9600
N3—C7	1.452 (4)	C6—H6A	0.9700
N3—C6	1.454 (3)	C6—H6B	0.9700
N3—C8	1.456 (3)	C7—H7A	0.9600
C1—C2^i^	1.394 (3)	C7—H7B	0.9600
C1—C3	1.506 (3)	C7—H7C	0.9600
C2—C6	1.505 (3)	C8—H8A	0.9600
C3—H3A	0.9700	C8—H8B	0.9600
C3—H3B	0.9700	C8—H8C	0.9600
C4—H4A	0.9600		
			
C2—N1—C1	117.5 (2)	N2—C5—H5C	109.5
C5—N2—C3	111.0 (2)	N2—C5—H5B	109.5
C5—N2—C4	110.8 (2)	H5C—C5—H5B	109.5
C3—N2—C4	111.3 (2)	N2—C5—H5A	109.5
C7—N3—C6	111.2 (2)	H5C—C5—H5A	109.5
C7—N3—C8	110.0 (2)	H5B—C5—H5A	109.5
C6—N3—C8	110.9 (2)	N3—C6—C2	112.3 (2)
N1—C1—C2^i^	121.0 (2)	N3—C6—H6A	109.1
N1—C1—C3	117.3 (2)	C2—C6—H6A	109.1
C2^i^—C1—C3	121.7 (2)	N3—C6—H6B	109.1
N1—C2—C1^i^	121.5 (2)	C2—C6—H6B	109.1
N1—C2—C6	116.5 (2)	H6A—C6—H6B	107.9
C1^i^—C2—C6	121.9 (2)	N3—C7—H7A	109.5
N2—C3—C1	111.3 (2)	N3—C7—H7B	109.5
N2—C3—H3A	109.4	H7A—C7—H7B	109.5
C1—C3—H3A	109.4	N3—C7—H7C	109.5
N2—C3—H3B	109.4	H7A—C7—H7C	109.5
C1—C3—H3B	109.4	H7B—C7—H7C	109.5
H3A—C3—H3B	108.0	N3—C8—H8A	109.5
N2—C4—H4A	109.5	N3—C8—H8B	109.5
N2—C4—H4B	109.5	H8A—C8—H8B	109.5
H4A—C4—H4B	109.5	N3—C8—H8C	109.5
N2—C4—H4C	109.5	H8A—C8—H8C	109.5
H4A—C4—H4C	109.5	H8B—C8—H8C	109.5
H4B—C4—H4C	109.5		
			
C2—N1—C1—C2^i^	−0.1 (3)	N1—C1—C3—N2	103.4 (2)
C2—N1—C1—C3	−178.68 (19)	C2^i^—C1—C3—N2	−75.2 (3)
C1—N1—C2—C1^i^	0.1 (3)	C7—N3—C6—C2	71.6 (3)
C1—N1—C2—C6	−179.79 (19)	C8—N3—C6—C2	−165.7 (2)
C5—N2—C3—C1	−77.0 (3)	N1—C2—C6—N3	−108.7 (2)
C4—N2—C3—C1	159.1 (2)	C1^i^—C2—C6—N3	71.4 (3)

Symmetry code: (i) −*x*, −*y*, −*z*+1.

### 1,1',1'',1'''-(Pyrazine-2,3,5,6-tetrayl)tetrakis(N,N-dimethylmethanamine) (I) Hydrogen-bond geometry (Å, º)

**Table:**

*D*—H···*A*	*D*—H	H···*A*	*D*···*A*	*D*—H···*A*
C3—H3*A*···N3^i^	0.97	2.62	3.261 (4)	124

Symmetry code: (i) −*x*, −*y*, −*z*+1.

### N,N',N'',N'''-[Pyrazine-2,3,5,6\ tetrayltetrakis(methylene)]tetrakis(N-methylaniline) (II) Crystal data

**Table:**

C_36_H_40_N_6_	*Z* = 1
*M**_r_* = 556.74	*F*(000) = 298
Triclinic, *P*1	*D*_x_ = 1.251 Mg m^−^^3^
*a* = 8.6753 (10) Å	Mo *K*α radiation, λ = 0.71073 Å
*b* = 8.9160 (11) Å	Cell parameters from 28 reflections
*c* = 10.0631 (10) Å	θ = 12.5–17.6°
α = 85.774 (10)°	µ = 0.08 mm^−^^1^
β = 73.468 (11)°	*T* = 293 K
γ = 82.467 (11)°	Block, pale-greenish-yellow
*V* = 739.21 (15) Å^3^	0.38 × 0.30 × 0.27 mm

### N,N',N'',N'''-[Pyrazine-2,3,5,6\ tetrayltetrakis(methylene)]tetrakis(N-methylaniline) (II) Data collection

**Table:**

Stoe–Siemens AED2, 4-circle diffractometer	*R*_int_ = 0.031
Radiation source: fine-focus sealed tube	θ_max_ = 25.5°, θ_min_ = 2.1°
Plane graphite monochromator	*h* = −9→10
ω/\2q scans	*k* = −10→10
5354 measured reflections	*l* = −12→12
2741 independent reflections	2 standard reflections every 60 min
1913 reflections with *I* > 2σ(*I*)	intensity decay: 1%

### N,N',N'',N'''-[Pyrazine-2,3,5,6\ tetrayltetrakis(methylene)]tetrakis(N-methylaniline) (II) Refinement

**Table:**

Refinement on *F*^2^	Secondary atom site location: difference Fourier map
Least-squares matrix: full	Hydrogen site location: inferred from neighbouring sites
*R*[*F*^2^ > 2σ(*F*^2^)] = 0.049	H-atom parameters constrained
*wR*(*F*^2^) = 0.108	*w* = 1/[σ^2^(*F*_o_^2^) + (0.0254*P*)^2^ + 0.254*P*] where *P* = (*F*_o_^2^ + 2*F*_c_^2^)/3
*S* = 1.12	(Δ/σ)_max_ < 0.001
2741 reflections	Δρ_max_ = 0.13 e Å^−^^3^
193 parameters	Δρ_min_ = −0.14 e Å^−^^3^
0 restraints	Extinction correction: (*SHELXL2018/3*; Sheldrick 2015), Fc^*^=kFc[1+0.001xFc^2^λ^3^/sin(2θ)]^-1/4^
Primary atom site location: structure-invariant direct methods	Extinction coefficient: 0.026 (3)

### N,N',N'',N'''-[Pyrazine-2,3,5,6\ tetrayltetrakis(methylene)]tetrakis(N-methylaniline) (II) Special details

**Table:**

Geometry. All esds (except the esd in the dihedral angle between two l.s. planes) are estimated using the full covariance matrix. The cell esds are taken into account individually in the estimation of esds in distances, angles and torsion angles; correlations between esds in cell parameters are only used when they are defined by crystal symmetry. An approximate (isotropic) treatment of cell esds is used for estimating esds involving l.s. planes.

### N,N',N'',N'''-[Pyrazine-2,3,5,6\ tetrayltetrakis(methylene)]tetrakis(N-methylaniline) (II) Fractional atomic coordinates and isotropic or equivalent isotropic displacement parameters (Å^2^)

**Table:**

	*x*	*y*	*z*	*U*_iso_*/*U*_eq_	
N1	0.46201 (19)	0.59971 (18)	0.89913 (16)	0.0393 (4)	
N2	0.5739 (2)	0.31235 (19)	0.66461 (17)	0.0454 (4)	
N3	0.2493 (2)	0.85914 (19)	0.95305 (17)	0.0481 (5)	
C1	0.5585 (2)	0.4702 (2)	0.86471 (19)	0.0366 (5)	
C2	0.4027 (2)	0.6301 (2)	1.0334 (2)	0.0378 (5)	
C3	0.6246 (2)	0.4442 (2)	0.7110 (2)	0.0437 (5)	
H3A	0.590818	0.533444	0.660508	0.052*	
H3B	0.741879	0.433034	0.687257	0.052*	
C4	0.4248 (3)	0.3268 (2)	0.63526 (19)	0.0429 (5)	
C5	0.3070 (3)	0.4493 (3)	0.6795 (2)	0.0502 (6)	
H5	0.328637	0.524582	0.728604	0.060*	
C6	0.1604 (3)	0.4603 (3)	0.6515 (2)	0.0619 (7)	
H6	0.083983	0.542794	0.682224	0.074*	
C7	0.1237 (3)	0.3508 (3)	0.5785 (3)	0.0674 (7)	
H7	0.024139	0.359185	0.559288	0.081*	
C8	0.2367 (3)	0.2306 (3)	0.5354 (3)	0.0661 (7)	
H8	0.212966	0.155880	0.486985	0.079*	
C9	0.3863 (3)	0.2167 (2)	0.5618 (2)	0.0549 (6)	
H9	0.461601	0.133665	0.530481	0.066*	
C10	0.7008 (3)	0.2013 (3)	0.5894 (3)	0.0645 (7)	
H10A	0.799406	0.208832	0.612583	0.097*	
H10B	0.717471	0.220396	0.491434	0.097*	
H10C	0.669345	0.101522	0.614401	0.097*	
C11	0.2938 (3)	0.7782 (2)	1.0686 (2)	0.0488 (6)	
H11A	0.195686	0.756971	1.138934	0.059*	
H11B	0.348395	0.843600	1.108603	0.059*	
C12	0.1177 (2)	0.8292 (2)	0.9122 (2)	0.0431 (5)	
C13	0.0121 (3)	0.7258 (3)	0.9845 (2)	0.0586 (6)	
H13	0.032849	0.671020	1.061037	0.070*	
C14	−0.1220 (3)	0.7040 (3)	0.9440 (3)	0.0697 (7)	
H14	−0.191144	0.635993	0.994996	0.084*	
C15	−0.1562 (3)	0.7802 (3)	0.8302 (3)	0.0690 (7)	
H15	−0.247073	0.765006	0.803564	0.083*	
C16	−0.0517 (3)	0.8795 (3)	0.7571 (3)	0.0643 (7)	
H16	−0.072129	0.931190	0.679059	0.077*	
C17	0.0822 (3)	0.9052 (2)	0.7956 (2)	0.0516 (6)	
H17	0.149960	0.973716	0.743706	0.062*	
C18	0.3667 (3)	0.9499 (3)	0.8637 (3)	0.0633 (7)	
H18A	0.407036	0.906772	0.774033	0.095*	
H18B	0.454775	0.952213	0.903352	0.095*	
H18C	0.316286	1.051147	0.854497	0.095*	

### N,N',N'',N'''-[Pyrazine-2,3,5,6\ tetrayltetrakis(methylene)]tetrakis(N-methylaniline) (II) Atomic displacement parameters (Å^2^)

**Table:**

	*U*^11^	*U*^22^	*U*^33^	*U*^12^	*U*^13^	*U*^23^
N1	0.0443 (10)	0.0408 (10)	0.0350 (9)	−0.0041 (8)	−0.0144 (7)	−0.0032 (7)
N2	0.0498 (11)	0.0481 (10)	0.0390 (10)	0.0057 (8)	−0.0163 (8)	−0.0112 (8)
N3	0.0541 (11)	0.0469 (11)	0.0455 (10)	0.0000 (9)	−0.0210 (9)	0.0016 (8)
C1	0.0371 (11)	0.0405 (11)	0.0347 (11)	−0.0066 (9)	−0.0126 (9)	−0.0044 (9)
C2	0.0400 (11)	0.0397 (11)	0.0366 (11)	−0.0044 (9)	−0.0147 (9)	−0.0040 (9)
C3	0.0454 (12)	0.0489 (12)	0.0360 (11)	−0.0029 (10)	−0.0106 (9)	−0.0039 (9)
C4	0.0523 (13)	0.0451 (12)	0.0314 (11)	−0.0027 (10)	−0.0142 (9)	0.0021 (9)
C5	0.0551 (14)	0.0576 (14)	0.0379 (12)	0.0042 (11)	−0.0159 (10)	−0.0098 (10)
C6	0.0490 (14)	0.0793 (18)	0.0521 (14)	0.0084 (13)	−0.0120 (11)	−0.0046 (13)
C7	0.0535 (15)	0.090 (2)	0.0637 (16)	−0.0169 (14)	−0.0239 (13)	0.0119 (15)
C8	0.0831 (19)	0.0582 (16)	0.0719 (17)	−0.0206 (14)	−0.0412 (15)	0.0038 (13)
C9	0.0747 (16)	0.0424 (13)	0.0524 (14)	−0.0010 (11)	−0.0275 (12)	−0.0030 (10)
C10	0.0631 (16)	0.0667 (16)	0.0613 (15)	0.0207 (13)	−0.0210 (12)	−0.0223 (13)
C11	0.0574 (14)	0.0506 (13)	0.0406 (12)	0.0052 (10)	−0.0210 (10)	−0.0074 (10)
C12	0.0464 (12)	0.0381 (11)	0.0429 (12)	0.0086 (9)	−0.0140 (10)	−0.0078 (9)
C13	0.0673 (16)	0.0563 (15)	0.0519 (14)	−0.0083 (12)	−0.0173 (12)	0.0048 (11)
C14	0.0597 (16)	0.0732 (18)	0.0723 (18)	−0.0179 (13)	−0.0071 (14)	−0.0057 (14)
C15	0.0550 (15)	0.0744 (18)	0.083 (2)	0.0020 (14)	−0.0280 (14)	−0.0203 (15)
C16	0.0704 (17)	0.0633 (16)	0.0654 (16)	0.0085 (13)	−0.0356 (14)	−0.0040 (13)
C17	0.0555 (14)	0.0492 (13)	0.0509 (13)	−0.0012 (11)	−0.0196 (11)	0.0026 (10)
C18	0.0677 (16)	0.0617 (15)	0.0676 (16)	−0.0143 (13)	−0.0287 (13)	0.0036 (13)

### N,N',N'',N'''-[Pyrazine-2,3,5,6\ tetrayltetrakis(methylene)]tetrakis(N-methylaniline) (II) Geometric parameters (Å, º)

**Table:**

N1—C2	1.336 (2)	C8—H8	0.9300
N1—C1	1.339 (2)	C9—H9	0.9300
N2—C4	1.395 (3)	C10—H10A	0.9600
N2—C10	1.454 (3)	C10—H10B	0.9600
N2—C3	1.459 (3)	C10—H10C	0.9600
N3—C12	1.382 (3)	C11—H11A	0.9700
N3—C11	1.442 (2)	C11—H11B	0.9700
N3—C18	1.445 (3)	C12—C13	1.400 (3)
C1—C2^i^	1.395 (3)	C12—C17	1.401 (3)
C1—C3	1.512 (3)	C13—C14	1.378 (3)
C2—C11	1.521 (3)	C13—H13	0.9300
C3—H3A	0.9700	C14—C15	1.374 (4)
C3—H3B	0.9700	C14—H14	0.9300
C4—C9	1.398 (3)	C15—C16	1.371 (4)
C4—C5	1.398 (3)	C15—H15	0.9300
C5—C6	1.369 (3)	C16—C17	1.375 (3)
C5—H5	0.9300	C16—H16	0.9300
C6—C7	1.384 (3)	C17—H17	0.9300
C6—H6	0.9300	C18—H18A	0.9600
C7—C8	1.359 (4)	C18—H18B	0.9600
C7—H7	0.9300	C18—H18C	0.9600
C8—C9	1.386 (3)		
			
C2—N1—C1	118.46 (16)	C7—C8—C9	121.7 (2)
C4—N2—C10	117.64 (17)	C7—C8—H8	119.2
C4—N2—C3	118.71 (16)	C9—C8—H8	119.2
C10—N2—C3	117.17 (18)	C8—C9—C4	120.4 (2)
C12—N3—C11	122.00 (18)	C8—C9—H9	119.8
C12—N3—C18	120.07 (17)	C4—C9—H9	119.8
C11—N3—C18	116.70 (18)	N3—C12—C13	122.77 (19)
N1—C1—C2^i^	120.79 (16)	N3—C12—C17	120.5 (2)
N1—C1—C3	115.85 (16)	C13—C12—C17	116.7 (2)
C2^i^—C1—C3	123.33 (17)	C14—C13—C12	121.0 (2)
N1—C2—C1^i^	120.74 (17)	C14—C13—H13	119.5
N1—C2—C11	117.03 (17)	C12—C13—H13	119.5
C1^i^—C2—C11	122.23 (17)	C15—C14—C13	121.6 (2)
N2—C3—C1	114.84 (17)	C15—C14—H14	119.2
N2—C3—H3A	108.6	C13—C14—H14	119.2
C1—C3—H3A	108.6	C16—C15—C14	117.7 (2)
N2—C3—H3B	108.6	C16—C15—H15	121.1
C1—C3—H3B	108.6	C14—C15—H15	121.1
H3A—C3—H3B	107.5	C15—C16—C17	122.1 (2)
N3—C11—C2	115.02 (17)	C15—C16—H16	119.0
N3—C11—H11A	108.5	C17—C16—H16	119.0
C2—C11—H11A	108.5	C16—C17—C12	120.8 (2)
N3—C11—H11B	108.5	C16—C17—H17	119.6
C2—C11—H11B	108.5	C12—C17—H17	119.6
H11A—C11—H11B	107.5	N3—C18—H18A	109.5
N2—C4—C9	120.81 (19)	N3—C18—H18B	109.5
N2—C4—C5	121.91 (19)	H18A—C18—H18B	109.5
C9—C4—C5	117.3 (2)	N3—C18—H18C	109.5
C6—C5—C4	121.1 (2)	H18A—C18—H18C	109.5
C6—C5—H5	119.5	H18B—C18—H18C	109.5
C4—C5—H5	119.5	N2—C10—H10A	109.5
C5—C6—C7	121.2 (2)	N2—C10—H10B	109.5
C5—C6—H6	119.4	H10A—C10—H10B	109.5
C7—C6—H6	119.4	N2—C10—H10C	109.5
C8—C7—C6	118.4 (2)	H10A—C10—H10C	109.5
C8—C7—H7	120.8	H10B—C10—H10C	109.5
C6—C7—H7	120.8		
			
C2—N1—C1—C2^i^	−0.5 (3)	C4—C5—C6—C7	−0.1 (4)
C2—N1—C1—C3	−178.66 (17)	C5—C6—C7—C8	0.4 (4)
C1—N1—C2—C1^i^	0.5 (3)	C6—C7—C8—C9	−0.6 (4)
C1—N1—C2—C11	179.92 (17)	C7—C8—C9—C4	0.5 (4)
C4—N2—C3—C1	83.8 (2)	N2—C4—C9—C8	178.8 (2)
C10—N2—C3—C1	−125.0 (2)	C5—C4—C9—C8	−0.2 (3)
N1—C1—C3—N2	−117.34 (19)	C11—N3—C12—C13	4.2 (3)
C2^i^—C1—C3—N2	64.5 (2)	C18—N3—C12—C13	171.1 (2)
C12—N3—C11—C2	86.2 (2)	C11—N3—C12—C17	−177.13 (19)
C18—N3—C11—C2	−81.1 (2)	C18—N3—C12—C17	−10.2 (3)
N1—C2—C11—N3	8.2 (3)	N3—C12—C13—C14	177.1 (2)
C1^i^—C2—C11—N3	−172.42 (18)	C17—C12—C13—C14	−1.7 (3)
C10—N2—C4—C9	14.5 (3)	C12—C13—C14—C15	1.2 (4)
C3—N2—C4—C9	165.50 (19)	C13—C14—C15—C16	0.1 (4)
C10—N2—C4—C5	−166.6 (2)	C14—C15—C16—C17	−0.8 (4)
C3—N2—C4—C5	−15.6 (3)	C15—C16—C17—C12	0.3 (4)
N2—C4—C5—C6	−178.9 (2)	N3—C12—C17—C16	−177.8 (2)
C9—C4—C5—C6	0.0 (3)	C13—C12—C17—C16	1.0 (3)

Symmetry code: (i) −*x*+1, −*y*+1, −*z*+2.

### N,N',N'',N'''-[Pyrazine-2,3,5,6\ tetrayltetrakis(methylene)]tetrakis(N-methylaniline) (II) Hydrogen-bond geometry (Å, º)

*Cg*2 and *Cg*3 are the centroids of rings C4–C9 and C12–C17, respectively.

**Table:**

*D*—H···*A*	*D*—H	H···*A*	*D*···*A*	*D*—H···*A*
C5—H5···N1	0.93	2.50	3.331 (3)	149
C6—H6···*Cg*3	0.93	2.99	3.804 (3)	147
C3—H3*A*···C*g*2^ii^	0.97	2.83	3.561 (2)	133

Symmetry code: (ii) −*x*+1, −*y*+1, −*z*+1.

### N,N'-[(6-Phenyl-6,7-dihydro-5H-pyrrolo[3,4-\ b]pyrazine-2,3-diyl)bis(methylene)]bis(N-methylaniline) (III) Crystal data

**Table:**

C_28_H_29_N_5_	*Z* = 2
*M**_r_* = 435.56	*F*(000) = 464
Triclinic, *P*1	*D*_x_ = 1.247 Mg m^−^^3^
*a* = 8.686 (1) Å	Mo *K*α radiation, λ = 0.71073 Å
*b* = 9.7731 (11) Å	Cell parameters from 5000 reflections
*c* = 14.3948 (16) Å	θ = 1.7–26.1°
α = 85.915 (13)°	µ = 0.08 mm^−^^1^
β = 75.349 (13)°	*T* = 293 K
γ = 78.891 (13)°	Hexagonal plate, pale yellow
*V* = 1159.8 (2) Å^3^	0.45 × 0.35 × 0.10 mm

### N,N'-[(6-Phenyl-6,7-dihydro-5H-pyrrolo[3,4-\ b]pyrazine-2,3-diyl)bis(methylene)]bis(N-methylaniline) (III) Data collection

**Table:**

Stoe IPDS 1 diffractometer	1518 reflections with *I* > 2σ(*I*)
Radiation source: fine-focus sealed tube	*R*_int_ = 0.051
Plane graphite monochromator	θ_max_ = 25.3°, θ_min_ = 2.1°
φ rotation scans	*h* = −10→10
8653 measured reflections	*k* = −11→11
3953 independent reflections	*l* = −17→17

### N,N'-[(6-Phenyl-6,7-dihydro-5H-pyrrolo[3,4-\ b]pyrazine-2,3-diyl)bis(methylene)]bis(N-methylaniline) (III) Refinement

**Table:**

Refinement on *F*^2^	Secondary atom site location: difference Fourier map
Least-squares matrix: full	Hydrogen site location: inferred from neighbouring sites
*R*[*F*^2^ > 2σ(*F*^2^)] = 0.035	H-atom parameters constrained
*wR*(*F*^2^) = 0.079	*w* = 1/[σ^2^(*F*_o_^2^) + (0.0308*P*)^2^] where *P* = (*F*_o_^2^ + 2*F*_c_^2^)/3
*S* = 0.68	(Δ/σ)_max_ < 0.001
3953 reflections	Δρ_max_ = 0.12 e Å^−^^3^
301 parameters	Δρ_min_ = −0.12 e Å^−^^3^
0 restraints	Extinction correction: (*SHELXL2018/3*; Sheldrick, 2015), Fc^*^=kFc[1+0.001xFc^2^λ^3^/sin(2θ)]^-1/4^
Primary atom site location: structure-invariant direct methods	Extinction coefficient: 0.0154 (11)

### N,N'-[(6-Phenyl-6,7-dihydro-5H-pyrrolo[3,4-\ b]pyrazine-2,3-diyl)bis(methylene)]bis(N-methylaniline) (III) Special details

**Table:**

Geometry. All esds (except the esd in the dihedral angle between two l.s. planes) are estimated using the full covariance matrix. The cell esds are taken into account individually in the estimation of esds in distances, angles and torsion angles; correlations between esds in cell parameters are only used when they are defined by crystal symmetry. An approximate (isotropic) treatment of cell esds is used for estimating esds involving l.s. planes.

### N,N'-[(6-Phenyl-6,7-dihydro-5H-pyrrolo[3,4-\ b]pyrazine-2,3-diyl)bis(methylene)]bis(N-methylaniline) (III) Fractional atomic coordinates and isotropic or equivalent isotropic displacement parameters (Å^2^)

**Table:**

	*x*	*y*	*z*	*U*_iso_*/*U*_eq_	
N1	0.4060 (2)	0.8786 (2)	0.20916 (11)	0.0559 (5)	
N2	0.2666 (2)	0.9790 (2)	0.05437 (12)	0.0596 (6)	
N3	0.5915 (2)	0.6863 (2)	−0.00687 (11)	0.0641 (6)	
N4	−0.0765 (2)	1.1354 (2)	0.19845 (12)	0.0567 (5)	
N5	0.2256 (2)	0.9508 (2)	0.39598 (12)	0.0607 (6)	
C1	0.2797 (3)	0.9865 (2)	0.21849 (14)	0.0536 (6)	
C2	0.4566 (2)	0.8223 (2)	0.12258 (14)	0.0510 (6)	
C3	0.3862 (3)	0.8699 (3)	0.04787 (13)	0.0522 (6)	
C4	0.2133 (2)	1.0385 (2)	0.14142 (15)	0.0550 (6)	
C5	0.5911 (3)	0.7029 (2)	0.09341 (13)	0.0611 (7)	
H5A	0.692953	0.724226	0.098842	0.073*	
H5B	0.570113	0.619743	0.131738	0.073*	
C6	0.4643 (2)	0.7825 (2)	−0.03875 (13)	0.0577 (7)	
H6A	0.388274	0.733319	−0.055210	0.069*	
H6B	0.508592	0.838726	−0.093682	0.069*	
C7	0.6870 (3)	0.5773 (3)	−0.06142 (15)	0.0559 (6)	
C8	0.8004 (3)	0.4812 (3)	−0.02614 (15)	0.0620 (7)	
H8	0.810894	0.489435	0.035832	0.074*	
C9	0.8976 (3)	0.3735 (3)	−0.08282 (17)	0.0725 (8)	
H9	0.972506	0.309893	−0.058215	0.087*	
C10	0.8853 (3)	0.3590 (3)	−0.17452 (19)	0.0773 (8)	
H10	0.951563	0.286567	−0.212066	0.093*	
C11	0.7738 (3)	0.4528 (3)	−0.21026 (16)	0.0764 (8)	
H11	0.764776	0.443527	−0.272410	0.092*	
C12	0.6751 (3)	0.5608 (3)	−0.15511 (15)	0.0665 (7)	
H12	0.599899	0.623164	−0.180367	0.080*	
C13	0.2210 (3)	1.0516 (2)	0.31746 (14)	0.0602 (7)	
H13A	0.287658	1.119063	0.321236	0.072*	
H13B	0.110758	1.101454	0.324780	0.072*	
C14	0.1121 (3)	0.8654 (3)	0.42226 (14)	0.0560 (6)	
C15	−0.0211 (3)	0.8838 (3)	0.38231 (15)	0.0629 (7)	
H15	−0.032502	0.953551	0.335842	0.076*	
C16	−0.1360 (3)	0.8000 (3)	0.41067 (19)	0.0781 (8)	
H16	−0.224404	0.814656	0.383465	0.094*	
C17	−0.1225 (4)	0.6956 (3)	0.4781 (2)	0.0906 (9)	
H17	−0.199630	0.638472	0.496365	0.109*	
C18	0.0080 (5)	0.6771 (3)	0.51850 (19)	0.0919 (10)	
H18	0.017561	0.607346	0.565218	0.110*	
C19	0.1240 (3)	0.7587 (3)	0.49158 (17)	0.0713 (8)	
H19	0.211426	0.743206	0.519603	0.086*	
C20	0.3718 (3)	0.9195 (3)	0.43102 (17)	0.0861 (9)	
H20A	0.435685	0.990627	0.408568	0.129*	
H20B	0.433210	0.830763	0.407665	0.129*	
H20C	0.343173	0.916359	0.499973	0.129*	
C21	0.0812 (3)	1.1664 (2)	0.14886 (15)	0.0651 (7)	
H21A	0.106550	1.237790	0.183089	0.078*	
H21B	0.077662	1.202914	0.084840	0.078*	
C22	−0.1951 (3)	1.2457 (3)	0.24527 (14)	0.0534 (6)	
C23	−0.1678 (3)	1.3796 (3)	0.24765 (15)	0.0688 (7)	
H23	−0.069134	1.402028	0.213889	0.083*	
C24	−0.2859 (4)	1.4813 (3)	0.29992 (19)	0.0860 (9)	
H24	−0.264807	1.570861	0.301192	0.103*	
C25	−0.4332 (4)	1.4517 (4)	0.34971 (19)	0.0920 (11)	
H25	−0.511567	1.519698	0.385288	0.110*	
C26	−0.4620 (3)	1.3199 (4)	0.34573 (18)	0.0911 (10)	
H26	−0.562113	1.299000	0.378099	0.109*	
C27	−0.3461 (3)	1.2173 (3)	0.29485 (16)	0.0719 (8)	
H27	−0.368766	1.128393	0.293559	0.086*	
C28	−0.1287 (3)	1.0303 (3)	0.15362 (17)	0.0805 (8)	
H28A	−0.200292	0.983359	0.201593	0.121*	
H28B	−0.036112	0.963950	0.123096	0.121*	
H28C	−0.184352	1.073996	0.106462	0.121*	

### N,N'-[(6-Phenyl-6,7-dihydro-5H-pyrrolo[3,4-\ b]pyrazine-2,3-diyl)bis(methylene)]bis(N-methylaniline) (III) Atomic displacement parameters (Å^2^)

**Table:**

	*U*^11^	*U*^22^	*U*^33^	*U*^12^	*U*^13^	*U*^23^
N1	0.0524 (11)	0.0660 (15)	0.0465 (11)	−0.0088 (11)	−0.0077 (9)	−0.0042 (9)
N2	0.0533 (12)	0.0728 (15)	0.0465 (11)	−0.0058 (11)	−0.0064 (9)	0.0024 (10)
N3	0.0620 (12)	0.0785 (16)	0.0446 (11)	0.0103 (12)	−0.0146 (9)	−0.0111 (10)
N4	0.0522 (12)	0.0551 (14)	0.0605 (11)	−0.0069 (11)	−0.0106 (10)	−0.0064 (10)
N5	0.0562 (13)	0.0813 (17)	0.0459 (11)	−0.0163 (12)	−0.0104 (10)	−0.0081 (10)
C1	0.0503 (14)	0.0603 (18)	0.0468 (13)	−0.0137 (13)	−0.0020 (11)	−0.0047 (11)
C2	0.0461 (13)	0.0629 (17)	0.0413 (13)	−0.0085 (13)	−0.0070 (11)	−0.0013 (12)
C3	0.0469 (13)	0.0650 (17)	0.0419 (13)	−0.0106 (13)	−0.0056 (11)	0.0000 (12)
C4	0.0498 (14)	0.0601 (17)	0.0505 (14)	−0.0082 (13)	−0.0063 (12)	0.0022 (12)
C5	0.0567 (14)	0.0749 (19)	0.0467 (13)	−0.0009 (14)	−0.0100 (11)	−0.0089 (12)
C6	0.0524 (14)	0.0736 (18)	0.0449 (12)	−0.0084 (13)	−0.0094 (11)	−0.0039 (12)
C7	0.0486 (14)	0.0627 (18)	0.0532 (14)	−0.0095 (14)	−0.0052 (11)	−0.0076 (12)
C8	0.0555 (14)	0.0709 (19)	0.0563 (14)	−0.0066 (15)	−0.0098 (12)	−0.0068 (13)
C9	0.0645 (17)	0.069 (2)	0.0777 (18)	−0.0034 (15)	−0.0112 (14)	−0.0085 (15)
C10	0.0739 (19)	0.074 (2)	0.0766 (18)	−0.0108 (17)	0.0001 (15)	−0.0247 (15)
C11	0.0800 (19)	0.088 (2)	0.0597 (15)	−0.0135 (18)	−0.0103 (14)	−0.0186 (15)
C12	0.0623 (16)	0.080 (2)	0.0562 (15)	−0.0069 (15)	−0.0135 (12)	−0.0119 (13)
C13	0.0618 (16)	0.0653 (18)	0.0513 (13)	−0.0143 (13)	−0.0053 (11)	−0.0109 (13)
C14	0.0589 (16)	0.0647 (19)	0.0397 (13)	−0.0072 (15)	−0.0032 (12)	−0.0143 (12)
C15	0.0598 (16)	0.074 (2)	0.0540 (14)	−0.0175 (15)	−0.0057 (13)	−0.0109 (13)
C16	0.070 (2)	0.085 (2)	0.0761 (18)	−0.0172 (18)	−0.0039 (15)	−0.0237 (17)
C17	0.092 (2)	0.079 (3)	0.092 (2)	−0.039 (2)	0.0180 (18)	−0.0243 (19)
C18	0.127 (3)	0.069 (2)	0.0706 (19)	−0.018 (2)	−0.006 (2)	−0.0067 (15)
C19	0.086 (2)	0.064 (2)	0.0615 (16)	−0.0086 (17)	−0.0154 (14)	−0.0066 (14)
C20	0.0670 (17)	0.122 (3)	0.0789 (17)	−0.0167 (17)	−0.0316 (14)	−0.0145 (16)
C21	0.0603 (16)	0.0617 (19)	0.0639 (14)	−0.0040 (14)	−0.0043 (12)	0.0023 (13)
C22	0.0536 (16)	0.0608 (19)	0.0442 (12)	−0.0012 (14)	−0.0162 (11)	0.0003 (12)
C23	0.0726 (17)	0.061 (2)	0.0673 (16)	−0.0041 (17)	−0.0117 (13)	−0.0061 (14)
C24	0.100 (2)	0.065 (2)	0.0893 (19)	0.0040 (19)	−0.0269 (18)	−0.0202 (16)
C25	0.081 (2)	0.110 (3)	0.0742 (19)	0.019 (2)	−0.0162 (17)	−0.0368 (19)
C26	0.0629 (18)	0.121 (3)	0.0784 (19)	0.003 (2)	−0.0059 (14)	−0.0256 (19)
C27	0.0598 (16)	0.080 (2)	0.0711 (15)	−0.0083 (16)	−0.0100 (14)	−0.0066 (14)
C28	0.0687 (17)	0.086 (2)	0.0927 (18)	−0.0024 (16)	−0.0318 (15)	−0.0313 (16)

### N,N'-[(6-Phenyl-6,7-dihydro-5H-pyrrolo[3,4-\ b]pyrazine-2,3-diyl)bis(methylene)]bis(N-methylaniline) (III) Geometric parameters (Å, º)

**Table:**

N1—C2	1.333 (2)	C12—H12	0.9300
N1—C1	1.354 (2)	C13—H13A	0.9700
N2—C3	1.328 (2)	C13—H13B	0.9700
N2—C4	1.352 (2)	C14—C15	1.394 (3)
N3—C7	1.374 (2)	C14—C19	1.398 (3)
N3—C6	1.453 (2)	C15—C16	1.376 (3)
N3—C5	1.463 (2)	C15—H15	0.9300
N4—C22	1.415 (3)	C16—C17	1.367 (4)
N4—C28	1.449 (3)	C16—H16	0.9300
N4—C21	1.453 (3)	C17—C18	1.376 (4)
N5—C14	1.376 (3)	C17—H17	0.9300
N5—C13	1.449 (3)	C18—C19	1.367 (4)
N5—C20	1.454 (3)	C18—H18	0.9300
C1—C4	1.397 (3)	C19—H19	0.9300
C1—C13	1.527 (3)	C20—H20A	0.9600
C2—C3	1.377 (2)	C20—H20B	0.9600
C2—C5	1.483 (3)	C20—H20C	0.9600
C3—C6	1.498 (3)	C21—H21A	0.9700
C4—C21	1.515 (3)	C21—H21B	0.9700
C5—H5A	0.9700	C22—C23	1.378 (3)
C5—H5B	0.9700	C22—C27	1.395 (3)
C6—H6A	0.9700	C23—C24	1.390 (3)
C6—H6B	0.9700	C23—H23	0.9300
C7—C8	1.392 (3)	C24—C25	1.373 (4)
C7—C12	1.401 (3)	C24—H24	0.9300
C8—C9	1.385 (3)	C25—C26	1.366 (4)
C8—H8	0.9300	C25—H25	0.9300
C9—C10	1.370 (3)	C26—C27	1.378 (3)
C9—H9	0.9300	C26—H26	0.9300
C10—C11	1.373 (3)	C27—H27	0.9300
C10—H10	0.9300	C28—H28A	0.9600
C11—C12	1.380 (3)	C28—H28B	0.9600
C11—H11	0.9300	C28—H28C	0.9600
			
C2—N1—C1	114.96 (16)	C1—C13—H13B	108.9
C3—N2—C4	115.23 (17)	H13A—C13—H13B	107.7
C7—N3—C6	122.57 (16)	N5—C14—C15	121.3 (2)
C7—N3—C5	123.11 (17)	N5—C14—C19	121.3 (2)
C6—N3—C5	113.64 (16)	C15—C14—C19	117.3 (3)
C22—N4—C28	118.35 (19)	C16—C15—C14	120.9 (3)
C22—N4—C21	117.9 (2)	C16—C15—H15	119.5
C28—N4—C21	114.48 (18)	C14—C15—H15	119.5
C14—N5—C13	120.75 (19)	C17—C16—C15	121.1 (3)
C14—N5—C20	119.8 (2)	C17—C16—H16	119.4
C13—N5—C20	117.9 (2)	C15—C16—H16	119.4
N1—C1—C4	122.07 (18)	C16—C17—C18	118.5 (3)
N1—C1—C13	115.34 (18)	C16—C17—H17	120.8
C4—C1—C13	122.5 (2)	C18—C17—H17	120.8
N1—C2—C3	122.80 (19)	C19—C18—C17	121.6 (3)
N1—C2—C5	125.96 (18)	C19—C18—H18	119.2
C3—C2—C5	111.24 (18)	C17—C18—H18	119.2
N2—C3—C2	123.17 (19)	C18—C19—C14	120.6 (3)
N2—C3—C6	126.31 (18)	C18—C19—H19	119.7
C2—C3—C6	110.52 (19)	C14—C19—H19	119.7
N2—C4—C1	121.65 (19)	N5—C20—H20A	109.5
N2—C4—C21	115.81 (19)	N5—C20—H20B	109.5
C1—C4—C21	122.52 (19)	H20A—C20—H20B	109.5
N3—C5—C2	102.26 (16)	N5—C20—H20C	109.5
N3—C5—H5A	111.3	H20A—C20—H20C	109.5
C2—C5—H5A	111.3	H20B—C20—H20C	109.5
N3—C5—H5B	111.3	N4—C21—C4	112.0 (2)
C2—C5—H5B	111.3	N4—C21—H21A	109.2
H5A—C5—H5B	109.2	C4—C21—H21A	109.2
N3—C6—C3	102.22 (16)	N4—C21—H21B	109.2
N3—C6—H6A	111.3	C4—C21—H21B	109.2
C3—C6—H6A	111.3	H21A—C21—H21B	107.9
N3—C6—H6B	111.3	C23—C22—C27	117.8 (2)
C3—C6—H6B	111.3	C23—C22—N4	123.5 (2)
H6A—C6—H6B	109.2	C27—C22—N4	118.7 (3)
N3—C7—C8	121.19 (19)	C22—C23—C24	120.7 (3)
N3—C7—C12	120.9 (2)	C22—C23—H23	119.6
C8—C7—C12	117.9 (2)	C24—C23—H23	119.6
C9—C8—C7	120.4 (2)	C25—C24—C23	121.0 (3)
C9—C8—H8	119.8	C25—C24—H24	119.5
C7—C8—H8	119.8	C23—C24—H24	119.5
C10—C9—C8	121.2 (2)	C26—C25—C24	118.4 (3)
C10—C9—H9	119.4	C26—C25—H25	120.8
C8—C9—H9	119.4	C24—C25—H25	120.8
C9—C10—C11	119.1 (2)	C25—C26—C27	121.5 (3)
C9—C10—H10	120.4	C25—C26—H26	119.3
C11—C10—H10	120.4	C27—C26—H26	119.3
C10—C11—C12	120.8 (2)	C26—C27—C22	120.6 (3)
C10—C11—H11	119.6	C26—C27—H27	119.7
C12—C11—H11	119.6	C22—C27—H27	119.7
C11—C12—C7	120.6 (2)	N4—C28—H28A	109.5
C11—C12—H12	119.7	N4—C28—H28B	109.5
C7—C12—H12	119.7	H28A—C28—H28B	109.5
N5—C13—C1	113.53 (19)	N4—C28—H28C	109.5
N5—C13—H13A	108.9	H28A—C28—H28C	109.5
C1—C13—H13A	108.9	H28B—C28—H28C	109.5
N5—C13—H13B	108.9		
			
C2—N1—C1—C4	−2.9 (3)	N3—C7—C12—C11	178.6 (2)
C2—N1—C1—C13	−180.0 (2)	C8—C7—C12—C11	−0.4 (3)
C1—N1—C2—C3	0.0 (3)	C14—N5—C13—C1	−75.0 (2)
C1—N1—C2—C5	−179.5 (2)	C20—N5—C13—C1	91.0 (3)
C4—N2—C3—C2	−2.3 (3)	N1—C1—C13—N5	−37.6 (3)
C4—N2—C3—C6	178.1 (2)	C4—C1—C13—N5	145.3 (2)
N1—C2—C3—N2	2.8 (3)	C13—N5—C14—C15	−7.6 (3)
C5—C2—C3—N2	−177.7 (2)	C20—N5—C14—C15	−173.29 (19)
N1—C2—C3—C6	−177.6 (2)	C13—N5—C14—C19	173.80 (19)
C5—C2—C3—C6	2.0 (3)	C20—N5—C14—C19	8.1 (3)
C3—N2—C4—C1	−0.6 (3)	N5—C14—C15—C16	−178.5 (2)
C3—N2—C4—C21	177.8 (2)	C19—C14—C15—C16	0.2 (3)
N1—C1—C4—N2	3.4 (3)	C14—C15—C16—C17	−0.6 (4)
C13—C1—C4—N2	−179.8 (2)	C15—C16—C17—C18	0.9 (4)
N1—C1—C4—C21	−174.9 (2)	C16—C17—C18—C19	−1.0 (4)
C13—C1—C4—C21	2.0 (3)	C17—C18—C19—C14	0.7 (4)
C7—N3—C5—C2	−173.1 (2)	N5—C14—C19—C18	178.4 (2)
C6—N3—C5—C2	−2.3 (2)	C15—C14—C19—C18	−0.3 (3)
N1—C2—C5—N3	179.7 (2)	C22—N4—C21—C4	154.45 (18)
C3—C2—C5—N3	0.1 (2)	C28—N4—C21—C4	−59.3 (2)
C7—N3—C6—C3	174.2 (2)	N2—C4—C21—N4	103.8 (2)
C5—N3—C6—C3	3.3 (2)	C1—C4—C21—N4	−77.8 (3)
N2—C3—C6—N3	176.5 (2)	C28—N4—C22—C23	−146.2 (2)
C2—C3—C6—N3	−3.2 (2)	C21—N4—C22—C23	−1.2 (3)
C6—N3—C7—C8	−175.5 (2)	C28—N4—C22—C27	36.3 (3)
C5—N3—C7—C8	−5.5 (3)	C21—N4—C22—C27	−178.72 (19)
C6—N3—C7—C12	5.6 (3)	C27—C22—C23—C24	1.6 (3)
C5—N3—C7—C12	175.6 (2)	N4—C22—C23—C24	−175.92 (19)
N3—C7—C8—C9	−178.8 (2)	C22—C23—C24—C25	−0.6 (4)
C12—C7—C8—C9	0.1 (3)	C23—C24—C25—C26	−0.8 (4)
C7—C8—C9—C10	0.2 (4)	C24—C25—C26—C27	1.3 (4)
C8—C9—C10—C11	−0.3 (4)	C25—C26—C27—C22	−0.3 (4)
C9—C10—C11—C12	0.1 (4)	C23—C22—C27—C26	−1.2 (3)
C10—C11—C12—C7	0.3 (4)	N4—C22—C27—C26	176.5 (2)

### N,N'-[(6-Phenyl-6,7-dihydro-5H-pyrrolo[3,4-\ b]pyrazine-2,3-diyl)bis(methylene)]bis(N-methylaniline) (III) Hydrogen-bond geometry (Å, º)

*Cg*2 and *Cg*3 are the centroids of rings N1/N2/C1–C4 and C7–C12, respectively.

**Table:**

*D*—H···*A*	*D*—H	H···*A*	*D*···*A*	*D*—H···*A*
C15—H15···N4	0.93	2.61	3.542 (3)	175
C28—H28*B*···N2	0.96	2.59	3.323 (3)	133
C6—H6*B*···*Cg*2^i^	0.97	2.82	3.601 (2)	138
C23—H23···*Cg*3^i^	0.93	2.97	3.881 (3)	168

Symmetry code: (i) −*x*+1, −*y*+2, −*z*.
